# Diffuse Cardiac Uptake Misdiagnosed as Cardiac Amyloidosis in Bone Scan

**DOI:** 10.3390/diagnostics13213342

**Published:** 2023-10-30

**Authors:** Yeongjoo Lee, Jaehyuk Jang, Sae Jung Na

**Affiliations:** 1 Department of Radiology, Uijeongbu St. Mary’s Hospital, College of Medicine, The Catholic University of Korea, Seoul 06591, Republic of Korea; soap2222@msn.com; 2 Division of Cardiology, Department of Internal Medicine, Uijeongbu St. Mary’s Hospital, College of Medicine, The Catholic University of Korea, Seoul 06591, Republic of Korea; let87@naver.com

**Keywords:** bone scan, ^99m^Tc-HDP, cardiac amyloidosis, iron overload

## Abstract

In this presented case, a 77-year-old woman with an implanted prosthesis and ongoing knee pain underwent a bone scan using ^99m^Tc-hydroxydiphosphonate (HDP) in suspicion for bone infection. An incidental finding from this scan revealed diffuse cardiac uptake, necessitating further diagnostic procedures to exclude the possibility of cardiac amyloidosis. In the subsequent ^99m^Tc-3,3-diphosphono-1,2-propanodicarboxylic acid (DPD) scan and SPECT images, no perceptible cardiac uptake was observed at all. Upon retrospective review of the patient’s medical records, she received 1000 mg of ferric carboxymaltose for iron-deficient anemia the day before the ^99m^Tc-HDP bone scan. Therefore, it was assumed that the diffuse and temporary cardiac activity was due to the transient iron overload. We present and share these bone scan images in order to avoid possible future misinterpretation of cardiac amyloidosis.

**Figure 1:**
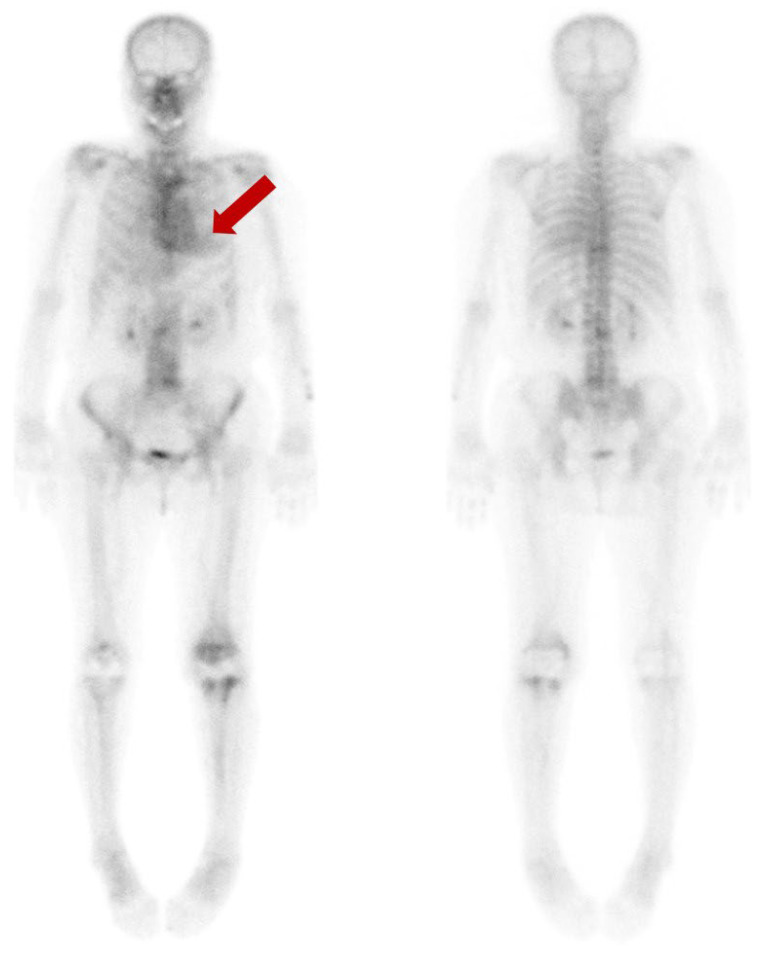
We present a 77-year-old woman who suffered ongoing left knee pain after the prosthesis insertion. To evaluate the extent and severity of the periprosthetic bone infection, a bone scan using ^99m^Tc-hydroxydiphosphonate (HDP) was performed. Beyond the expected uptake in the left knee’s periprosthetic region, an incidental finding indicated a diffuse and moderate cardiac uptake (arrow). This finding was classified as grade 2, based on the visual grading system ranging from 0 to 3, proposed by Perugini for the diagnosis of cardiac amyloidosis in bone scan [1].

**Figure 2:**
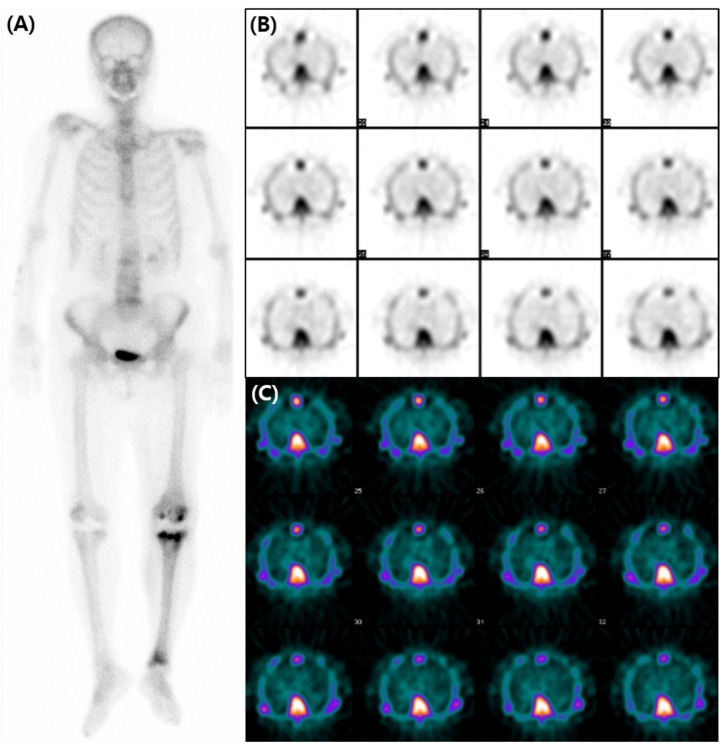
A subsequent bone scan using ^99m^Tc-3,3-diphosphono-1,2-propanodicarboxylic acid (DPD) was obtained three weeks later including SPECT images for assessment of transthyretin-type cardiac amyloidosis (CA). The decision was influenced by the accumulating evidence that favored the use of ^99m^Tc-DPD over ^99m^Tc-HDP for detecting CA [2,3,4], and by the necessity of SPECT images. However, no perceptible radiotracer accumulation was observed in the subsequent ^99m^Tc-DPD scan (**A**). Additionally, no perceptible radiotracer uptake was noted in the myocardium on the axial SPECT images (**B**,**C**). An echocardiogram revealed preserved ejection fraction of 60% without any abnormal wall thickness or abnormal wall motion. No abnormality was found in serum free light chain, serum and urine protein electrophoresis/immunofixation data. Upon thorough review of her medical history, her hemoglobin level was 8.7 g/dL, and she was diagnosed with iron-deficient anemia. We also observed that a total of 1000mg of ferric carboxymaltose had been intravenously administered the day before the ^99m^Tc-HDP bone scan. Hence, it is assumed that the diffuse and temporary cardiac activity was a result of transient iron overload. Bone scan using ^99m^Tc-DPD or ^99m^Tc-PYP is known for its several advantages in the diagnosis in CA. They not only have high sensitivity and specificity but also provide a non-invasive, whole-body evaluation and are readily accessible [5,6]. Currently, multidisciplinary experts in cardiovascular imaging and cardiac amyloidosis recommend including bone scans for diagnosis of CA [7]. However, caution should be exercised in the interpretation of myocardial uptake in a bone scan, as altered biodistribution of radiotracer can occur in various medical conditions including iron overload [8,9,10,11,12]. As bone scans are widely used in the diagnosis of CA, we present and share this image in order to avoid possible future misinterpretation.

## Data Availability

The data presented in this study are available upon request from the corresponding author.
